# Usage of *Chlorella* and diverse microalgae for CO_2_ capture - towards a bioenergy revolution

**DOI:** 10.3389/fbioe.2024.1387519

**Published:** 2024-08-20

**Authors:** Mohamed Ashour, Abdallah Tageldein Mansour, Yousef A. Alkhamis, Mostafa Elshobary

**Affiliations:** ^1^ National Institute of Oceanography and Fisheries (NIOF), Cairo, Egypt; ^2^ Animal and Fish Production Department, College of Agricultural and Food Sciences, King Faisal University, Al-Ahsa, Saudi Arabia; ^3^ Department of Fish and Animal Production, Faculty of Agriculture (Saba Basha), Alexandria University, Alexandria, Egypt; ^4^ Water and Environment Study Center, King Faisal University, Al-Ahsa, Saudi Arabia; ^5^ Department of Botany and microbiology, Faculty of Science, Tanta University, Tanta, Egypt

**Keywords:** Chlorella, climate change, CO_2_ fixation, carbon concentration mechanism, algal biomass, flue gas

## Abstract

To address climate change threats to ecosystems and the global economy, sustainable solutions for reducing atmospheric carbon dioxide (CO_2_) levels are crucial. Existing CO_2_ capture projects face challenges like high costs and environmental risks. This review explores leveraging microalgae, specifically the *Chlorella* genus, for CO_2_ capture and conversion into valuable bioenergy products like biohydrogen. The introduction section provides an overview of carbon pathways in microalgal cells and their role in CO_2_ capture for biomass production. It discusses current carbon credit industries and projects, highlighting the *Chlorella* genus’s carbon concentration mechanism (CCM) model for efficient CO_2_ sequestration. Factors influencing microalgal CO_2_ sequestration are examined, including pretreatment, pH, temperature, irradiation, nutrients, dissolved oxygen, and sources and concentrations of CO_2_. The review explores microalgae as a feedstock for various bioenergy applications like biodiesel, biooil, bioethanol, biogas and biohydrogen production. Strategies for optimizing biohydrogen yield from *Chlorella* are highlighted. Outlining the possibilities of further optimizations the review concludes by suggesting that microalgae and *Chlorella*-based CO_2_ capture is promising and offers contributions to achieve global climate goals.

## 1 Introduction

Global warming and greenhouse gas emissions significantly affect world energy, sustainability, and development (Lokuge and Anders, 2022). Climate change is a major threat that hinders the survival of various plants, animals, and human progress, as well as the wellbeing of our planet. The increased emissions of various greenhouse gases (GHGs), such as carbon dioxide (CO_2_), methane (CH_4_), nitrous oxide (N_2_O), sulfur dioxide (SO_2_), and fluorinated gases have worsened current climate changes, emphasizing the need to reduce CO_2_ emissions and promote the use of renewable sources, particularly fuels (Adams and Engel, 2021). Globally, CO_2_ accounts for 76% of total GHGs, mostly (72%) released by the energy production sector. In 2019, it reached approximately 33 gigatons. In the first quarter of 2020 compared to the first quarter of 2019, global CO_2_ emissions decreased by 5% due to a decline in the demand for coal, oil, and natural gas (8%, 4.5%, and 2.3%, respectively). This decrease in CO_2_ emissions in 2020 was largely caused by the COVID-19 pandemic, the largest decline since World War II (Nguyen et al., 2021).

The amount and concentration of CO_2_ vary depending on the source of the emission. For instance, flue gas of power plants is about 3%–4%, while coal-fired plants emit about 10%–13%. CO_2_ from bio-refineries can reach up to 80% (Prasad et al., 2021). Globally, atmospheric CO_2_ has increased from 313 ppm in 1960 to 411 ppm in 2020 and is projected to reach 450 ppm by 2035 (Barakat et al., 2021). Some scenarios predict an increase of up to 700 ppm in the future, which would result in a 99% probability of a 2 C rise in global warming and significant damage to the global economy (van Leeuwen et al., 2024). The reduction of CO_2_ emissions is a top concern for the world. It is essential to develop a plan to lower or stabilize CO_2_ levels in the atmosphere. Many countries have committed to reducing greenhouse gas emissions through international agreements such as the Kyoto Protocol (1997) and the Paris Agreement (2015). According to a study by Prasad et al. (2021), there are two main approaches to reducing CO_2_ emissions: i) decreasing the consumption of fossil fuels by increasing the use of renewable energy sources, and ii) capturing and storing CO_2_ through various biological, chemical, or physical methods. Osman et al. (2021) identified three primary strategies for CO_2_ capture, storage, and utilization: pre-combustion, post-combustion, and oxyfuel combustion technologies. Although significant research has been conducted on how to reduce CO_2_ emissions through physical and chemical means, there are numerous limitations, including environmental, technical, and economic factors. It is acknowledged that the scope of Carbon Capture and Utilization (CCU) technologies that directly use captured CO2 in industrial processes is limited and their impact on reducing emissions is minimal (Shreyash et al., 2021). Accordingly, it is essential to find appropriate, sustainable, and profitable approaches for capturing CO_2_ that reduce atmospheric CO_2_ levels more effectively than physical and chemical methods (Prasad et al., 2021).

Among CO_2_ capture, utilization, and storage technologies (CCUS), biological CCUS is the most economical and environmentally friendly option, relying mainly on sunlight and photosynthetic organisms such as aquatic and terrestrial plants (Nunez, 2019). The sun provides nearly infinite energy, with our planet receiving 100,000 terawatts annually compared to our current energy consumption of 15 terawatts, which is expected to increase to 24 TW y^−1^ by 2030 and 45 terawatts by the end of the century. It is a huge amount of energy compared to our current energy consumption (Benedetti et al., 2018). Although our current energy consumption is 15 TW y^−1^, and it is expected to increase to about 24 TW y^−1^ by 2030, and 45 TW y^−1^ by the end of this century, the energy received from the sun is more than 2,200 times that of our energy consumption (Gerotto et al., 2020). Photoautotrophic organisms convert CO_2_ into carbon-based compounds including sugars, proteins, and lipids with the use of water and sunlight (Ashour and Omran, 2022). Worldwide, photoautotrophic organisms, both aquatic and terrestrial plants, can store solar energy at a rate of 120 terawatts every year (Benedetti et al., 2018). That means that the annual capacity of photoautotrophic organisms to store energy in photosynthetic products exceeds the current global energy demand by 800%. Therefore, the extensive culture of these organisms is an important potential solution to cover a large part of world energy demand (Stephenson et al., 2011).

**FIGURE 1:**
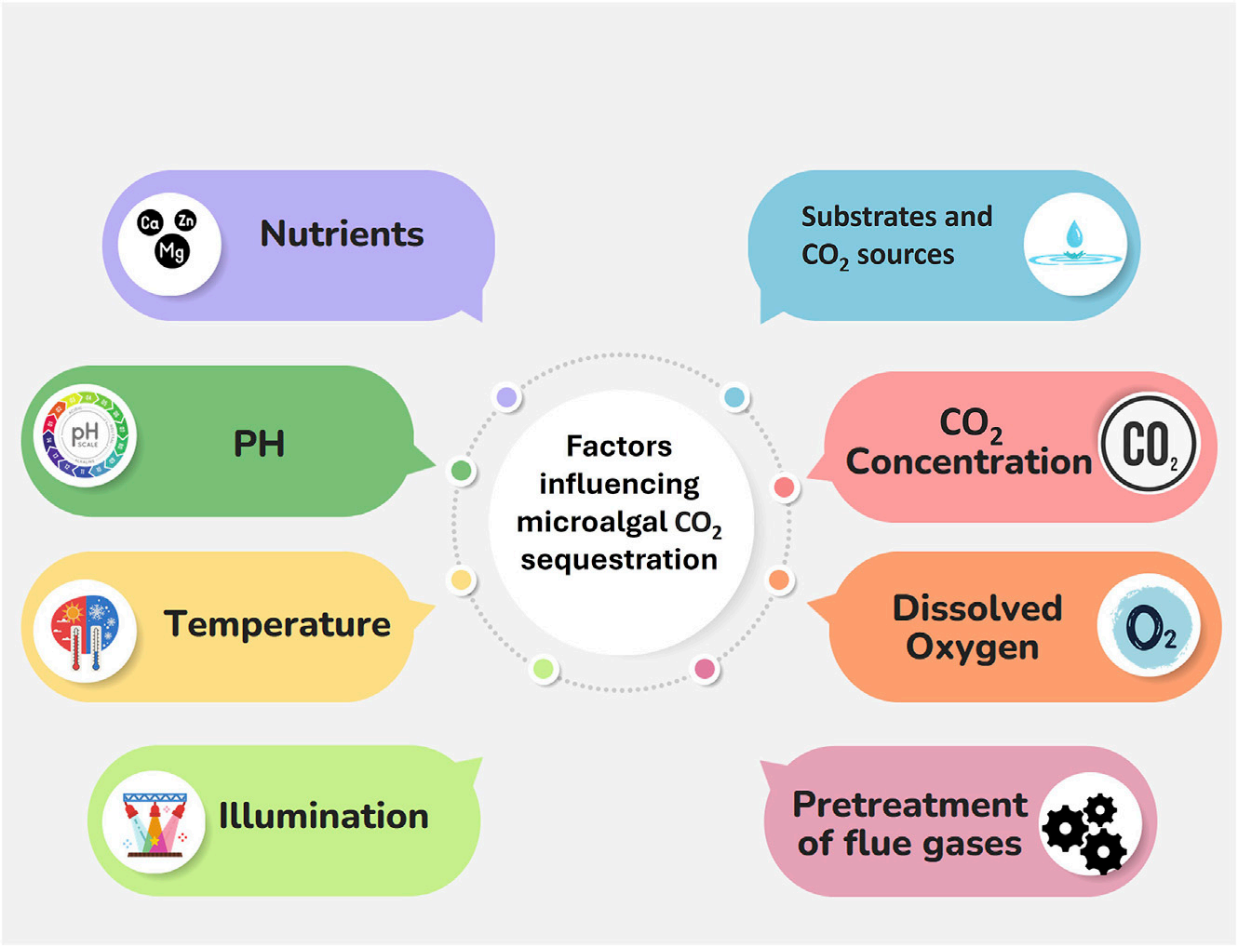
Factors affecting microalgal CO_2_ sequestration.

Among biological CCUS options, microalgae systems have emerged as a particularly promising route for atmospheric CO_2_ capture due to their high efficiency, scalability, and potential to generate valuable co-products. Through the process of photosynthesis, photoautotrophic organisms consume atmospheric CO_2_ and convert it into useful biomass, food, and bioactive compounds that are valuable in various industries. Despite their slow growth rate, terrestrial plants’ ability to capture CO_2_ is estimated to contribute only 3%–6% of fossil fuel emissions (Birner et al., 2023). In contrast, the faster growth rate of microalgae allows them to fix CO_2_ at a rate 10–50 times higher than that of terrestrial plants (Figure 1).

Nunez (2019) identifies various strategies to decrease global CO_2_ emissions, including widespread microalgae cultivation (especially in photobioreactors), tree planting, preserving grasslands and forests, improving energy efficiency, and boosting clean energy production. The terrestrial plants store energy mainly in the form of lignocellulose, a complex biopolymer that is challenging to utilize as a sustainable renewable feedstock. Aquatic plants, on the other hand, do not contain lignin, which makes it simple to use them in a variety of applications without requiring lengthy processing (Osman et al., 2023a). Additionally, the simultaneous synthesis of beneficial bioproducts such as lipids, proteins, carbohydrates, pigments, vitamins, and polyunsaturated fatty acids is possible in large quantities in aquatic plants. This multi-benefit of microalgae has garnered increasing interest in the field, as highlighted by the growing attention to microalgal CO_2_ bio-fixation and resource utilization in recent years (Lim et al., 2021). The multifaceted nature of these bio-products underscores the potential for comprehensive and sustainable applications in various industrial sectors.

Fixation of CO_2_ from the atmosphere by photoautotrophic organisms is achieved through the C3 and C4 pathways. Nevertheless, some microalgae have been found to have a higher capacity for CO_2_ capture compared to terrestrial C4 plants (Hong, 2022). CO_2_ capture projects by microalgae are currently viewed as highly attractive to investors for several reasons. According to the statistics report of the Food and Agriculture Organization (Cai et al., 2021), the global microalgae biomass production was 56,465 tons in 2019, with China accounting for 97.16% of the production, followed by Chile (1.6%), France (0.37%), Greece (0.25%), Tunisia (0.25%), Burkina Faso (0.25%), Central African Republic (0.09%), Chad (0.04%), Bulgaria (0.005%), and Spain (0.003%). Spirulina (*Arthrospira* sp.) represents 96.56% of the global microalgae biomass production, while *Haematococcus pluvialis* (0.429%), *Chlorella vulgaris* (0.008%), *Tetraselmis* sp. (0.003%), and *Dunaliella salina* (0.0004%) contributed the rest (Cai et al., 2021).

Microalgae contribute more than 90% of the primary production in marine ecosystems and fix about 50 gigatons of CO_2_ annually (Sommer et al., 2002). According to the study Kadam (2001), a 1,000-ha microalgae open pond (raceway) system could reduce CO_2_ emissions from flue gases by 50%, or 2,10,000 tons y^−1^ out of the 4,14,000 tons produced by a 50 megawatts power station. The CO_2_ absorbed by microalgae is converted into high-nutritional and economically valuable organic bioactive compounds (Zhou et al., 2022). As a result, algae industries have attracted the attention of investors worldwide for the potential use of algae for CO_2_ fixation and absorption (Zhang and Liu, 2021) and for using its biomass as raw material for various bioindustries, such as aqua-feed, biofertilizers, bioenergy, human food supplements and pharmaceuticals, and wastewater treatment (Mansour et al., 2022a; Mansour et al., 2022b; Alprol et al., 2023; Ashour et al., 2023; Elshobary and Ashour, 2023). Microalgae have attractive CO_2_ capture potential and high biomass productivity. Microalgae have a CO_2_ capture potential of 1.6–2 tons per year and a biomass productivity of 127–300 tons per hectare per year. The biomass productivity of microalgae can be significantly increased with advanced culture, harvest, and, drying technologies (Bai et al., 2017). The review by Klinthong et al. (2015) concluded that the increasing global interest in microalgae for CO_2_ capture and the production of various renewable energies is due to several advantages over terrestrial plants. These advantages include 1) high fixation of atmospheric CO_2_, 2) high conversion rate of the photosynthetic process, 3) rapid growth and production rate, 4) high potential for environmental phytoremediation, 5) capacity to produce various biomass and bioenergy resources, and 6) no competition with food and agricultural products. Thus, these advantages make microalgae a promising solution to reduce atmospheric CO_2_ levels and produce bioenergy (Klinthong et al., 2015). With microalgae emerging as prime candidates for efficient and scalable biological CO_2_ capture, ongoing research is quantifying the real-world potential of large-scale microalgae cultivation systems. A recent techno-economic evaluated various cultivation and harvesting scenarios, capturing 102.13 tons CO_2_/year/ha with operating costs ranging from $4.75–6.55/kg dry biomass (Valdovinos-García et al., 2020).

In alignment with the overarching objective of exploring CO_2_ mitigation strategies, this review discusses microalgae as a promising renewable feedstock for producing sustainable biofuels and other bioproducts due to their high biomass productivity, ability to utilize CO_2_, and potential for integration into biorefinery systems. The review will also include research on the usage of microalgae to produce renewable biohydrogen. The allure of biohydrogen lies in its potential as an energy-dense transportation fuel, poised to deliver substantial offsets in CO_2_ emissions while simultaneously fostering the generation of sustainable energy. As the examination continues, this review also examines current carbon credit projects, providing an overview of their feasibility in achieving global climate goals by 2050. The novelty of this study lies in its focus on utilizing microalgae for renewable bioenergy production, especially biohydrogen, as a sustainable and carbon-neutral energy source, thereby mitigating CO_2_ emissions and fostering clean energy generation.

## 2 Carbon pathways in microalgal cells

Microalgae have unique carbon pathways that enable them to take in carbon dioxide and produce oxygen through photosynthesis. Studying these pathways can provide valuable information on the functioning of aquatic plant projects in CO_2_ capture. Photosynthetic cells exchange CO_2_ and oxygen (O_2_) through their cell walls during photosynthesis. To assess the efficacy of capturing atmospheric CO_2_ into microalgal cells, it is crucial to examine their carbon pathways (Gehl et al., 1987). Microalgae capture approximately 50 gigatons of CO_2_ from the atmosphere annually, accounting for more than 50% of all photosynthetic activity worldwide. However, microalgae face three challenges in capturing and fixing CO_2_, as described by the study by Moroney and Ynalvez (2007). First, the enzyme Rubisco, which plays a crucial role in photosynthesis, has a poor CO_2_ affinity and operates at only 25% of its catalytic capacity due to the lower concentration of dissolved CO_2_ and the competition with O_2_ at atmospheric CO_2_ levels. Secondly, CO_2_ diffuses much slower in water compared to the atmosphere. Therefore, microalgae greatly benefit from the capacity to scavenge CO_2_ as soon as it becomes accessible. Lastly, the levels of inorganic carbon (C_i_ = CO_2_ + HCO_3_) and pH in the microalgal environment have a significant impact on the availability of CO_2_ and HCO_3_- for photosynthesis. When the pH is acidic, most of the available Ci is in the form of CO_2_, but when the pH is alkaline, the majority of the C_i_ is in the form of HCO_3_^─^ (Gehl et al., 1987).

Microalgae, as single-celled photosynthetic organisms, have evolved a specialized pathway to overcome the challenges associated with capturing and fixing atmospheric CO_2_ through photosynthesis. This pathway is called the Carbon Concentration Mechanism (CCM), and it resembles the C4 and Crassulacean Acid Metabolism (CAM) pathways found in terrestrial plants. The CCM increases the concentration of inorganic carbon several times over the level found in the surrounding environment, thereby enhancing the photosynthetic output of algal cells. To achieve this, microalgae have a specialized plastid structure called pyrenoid, which elevates the CO_2_ concentration around the thylakoid membranes. This in turn increases the efficiency of the Rubisco enzyme for carbon sequestration and assimilation. The CCM is a unique and innovative adaptation developed by microalgae to increase their ability to absorb and convert atmospheric CO_2_ into biomass (Barrett et al., 2021).

Interestingly, Carbonic Anhydrase (CA) is a zinc-containing metallic enzyme that has been found to play a significant part in the CCM and assist in the fixation of atmospheric CO_2_ by catalyzing the reversible hydration of CO_2_ into bicarbonate and a proton. CA assists CO_2_ fixation by nucleophilic attack by the hydroxide ion that is bound to a zinc atom. CA plays an important role in CO_2_ acquisition, capture, ion exchange, and photosynthesis. This reaction is followed by the removal of a proton from the protein surface and the ionization of the water molecule linked to zinc, which regenerates the active site. Therefore, the CA task in the fixation of carbon is to transform bicarbonate into CO_2_, which serves as the substrate for Rubisco, the main enzyme responsible for fixing carbon (Sayre, 2010).

As reported by the study by Prasad et al. (2021) photorespiration causes a loss of energy and carbon, ultimately lowering the rates of photosynthesis. Atmospheric O_2_ levels strongly exceed the CO_2_ concentration thus enhancing Rubisco´s oxigenase activity and the subsequent photorespiration. To combat this condition, microalgae have created CO_2_ concentration mechanisms (CCMs) to increase the levels of CO_2_ around Rubisco. Several studies have demonstrated different CCM strategies in several microalgae species.

Microalgae have developed CCMs as an adaption to increase the photosynthetic efficiency at low CO_2_ (Sayre, 2010; Moroney et al., 2013), thus allowing higher growth rates compared to terrestrial plants. As previously reported by the studies by Sayre, (2010); Giordano et al., (2005); Moroney and Ynalvez, (2007), the CCM is mainly based on the C4 and CAM pathways, in which PEP absorbs CO_2_ to produce oxalic-acetic acid (OAA). To maximize the capture of CO_2_, this pathway also enables to capture the CO_2_ produced during photorespiration (Giordano et al., 2005).

However, the CCM strategies differ among various microalgae species. For instance, Chlorella vulgaris utilizes a relatively simpler CCM compared to species like Chlamydomonas reinhardtii and *Nannochloropsis oceanica*, which have more complex mechanisms involving multiple carbon fixation pathways (Wei et al., 2019). These differences highlight the diversity in CCM strategies among microalgae and their varying efficiencies in CO_2_ capture and biomass production (Yu et al., 2020).

In a recent study Treves et al. (2022) investigated the ^13^CO_2_ *in vivo* labeling kinetics of the Calvin Benson Cycle and the pathways of organic acid, starch, sugar, amino acid, lipid, and protein synthesis in three green microalgae: *Chlorella sorokiniana, Chlorella ohadii,* and *Chlamydomonas reinhardtii*. The study also compared the flow patterns in these algal species with data from the C3 and C4 pathways from terrestrial plants. The findings showed unique flow patterns in these microalgae, which resulted in faster autotrophic growth. Furthermore, some species exhibited faster Rubisco regeneration and increased fluxes through reduced glycolysis and anaplerotic pathways to the tricarboxylic acid cycle, lipid synthesis, and amino acid synthesis compared to terrestrial plants. According to the literature, one of the highly-efficient green microalga, *Chlorella vulgaris*, demonstrated these enhanced metabolic fluxes. Genome-scale models suggest that during mixotrophic culture, there is increased carbon dioxide transport between the plastid and mitochondria in *Chlorella vulgaris*, accompanied by a 25% and 60% rise in the activity of carbon metabolism subsystems during mixotrophy and heterotrophy, respectively (Zuñiga et al., 2018). Similarly, *Chlorella protothecioides* exhibits heightened intracellular metabolite concentrations related to enhanced glycolysis and tricarboxylic acid cycle (TCA) activity during heterotrophy (Wu et al., 2015). These elevated TCA activities are associated with increased synthesis of storage compounds like fatty acids and carbohydrates (Vitova et al., 2015), along with species-specific changes in biochemical profiles (Penhaul Smith et al., 2021). The strain ZJU9000 is a stable mutant culture of *Arthrospira platensis*, produced through 9 kGy gamma irradiation, and exhibits improved growth compared to the wild-type. The study by Cheng et al. (2018) investigated differences in gene expression between wild-type and ZJU9000 and found that the robust growth of the mutation was due to higher levels of pigment and vitamin production, which improved photosynthesis and cell development. The study also revealed that ZJU9000 had higher CO_2_ capture at low concentrations compared to the wild-type, due to its enhanced CCM (Cheng et al., 2018). These findings were similar to those of other blue-green species like *Anabaena sp*. and *Microcystis aeruginosa*.

Among microalgae, marine diatoms are responsible for about 20% of world CO_2_ fixation (Khan et al., 2009). Like C4 plants, diatom species contain CCMs that utilize biochemical fixation of bicarbonate, but whether the same type of CCM is present in all diatoms is a subject of debate (Yu et al., 2022). The Phosphoenolpyruvate carboxylase enzyme (PEPcase enzyme, the primary enzyme in C4 and CAM pathways) is detected in marine diatom species in two isoforms, one in the plastids (PEPC1) and the other one in the mitochondria (PEPC2). The study by Yu et al. (2022) used several techniques (Western blots, real-time quantitative polymerase chain reaction, and enzymatic assays) to examine the expression and activities of PEPC1 and PEPC2 in *Phaeodactylum tricornutum*, under several concentrations of dissolved inorganic carbon (low and high). They generated and analyzed individual cell lines of both PEPC1 and PEPC2 of *P. tricornutum* and also generated and analyzed a double-knockout strain of PEPC1/2. Their findings implemented that, at least some of the CCM in the marine species *P. tricornutum* depends on the biochemical fixation of bicarbonate that is performed by the mitochondrial form of PEPC2 (Yu et al., 2022).

Dinoflagellates are significant primary producers and a main reason for harmful-toxic algal blooms in the marine ecosystems (Elshobary et al., 2020). The capture of carbon by dinoflagellates is still poorly understood, despite its enormous ecological importance (Carnicer et al., 2022). In the study by Zhang et al. (2021), the pathway of carbon capture in a marine dinoflagellate *Prorocentrum donghaiense*, including *in situ* and laboratory-simulated bloom conditions, were examined by using several techniques. They observed rapid capture of dissolved CO_2_ to produce high biomass during bloom. The genes responsible for CO_2_ capture were highly expressed at low levels of CO_2_, concluding that the C4 pathway exists in the blooming cells of *P. donghaiense*. Finally, they concluded that the C4 pathway in this marine dinoflagellate exhibited an important integrated function to assist the capture of CO_2_ during the bloom.

As reported in the study by Klinthong et al. (2015), the possible pathways of inorganic carbon in microalgae are: (1) direct CO_2_ capture through the plasma membrane; (2) the use of HCO_3_^─^ through activating the CA enzyme that converts HCO_3_^─^ to CO_2_, and (3) direct transport of HCO_3_^─^ through the plasma membrane. The different carbon assimilation pathways in some microalgae species are listed in Table 1.

**TABLE 1:** Carbon assimilation pathways as reported for selected microalgae^a^.

Microalgae species	Pathway 1 (Direct CO_2_ capture)	Pathway 2 (CA activation)	Pathway 3 (Direct HCO_3_^─^ capture)	References
Chlorella saccharophila	E	E	E	Rotatore and Colman (1991)
Chlorella ellipsoidea	E	M	E	Rotatore and Colman (1991)
Chlorella kesslerii	E	M	E	Bozzo et al. (2000)
Chlamydomonas reinhardtii	E	F	E	Sultemeyer et al. (1989)
Nannochloropsis gaditana	M	M	E	Huertas et al. (2000b)
Nannochloropsis oculata	M	M	E	Huertas et al. (2000b)
Nannochloris atomus	E	M	M	Huertas et al. (2000a)
Nannochloris maculata	E	M	M	Huertas et al. (2000a)
Dunaliella terteolecta	E	E	E	Amoroso et al. (1998)
Scenedesmus obliquus	E	E	E	Palmqvist et al. (1994)
Isochrysis galbana	E	E	E	Huertas et al. (2002)
Phaeodactylum tricornutum	E	E	E	Colman and Rotatore (1995)
Navicula pelliculosa	E	M	E	Rotatore and Colman (1992)
Cyclotella sp	E	E	E	Rotatore et al. (1995)
Ditylum brightwellii	E	M	E	Korb et al. (1997)
Skeletonema costatum	E	M	E	Korb et al. (1997)
Chaetoceros calcitrans	E	M	E	Korb et al. (1997)
Thalassiosira punctigera	E	M	NR	Elzenga et al. (2000)
Thalassiosira pseudonanna	NR	M	E	Elzenga et al. (2000)
Porphyridium cruentum	E	E	E	Colman and Rotatore (1995)
Emiliania huxleyi	E	E	NR	Elzenga et al. (2000)
Dicrateria inornata	E	E	E	Huertas et al. (2002)
Phaeocystis globosa	E	E	NR	Elzenga et al. (2000)
Vischeria stellata	E	M	E	Huertas et al. (2002)
Eremosphaera viridis	E	M	M	Rotatore and Colman (1992)
Amphidinium carterae	E	M	M	Huertas et al. (2002)
Heterocapsa oceanica	E	M	M	Huertas et al. (2002)
Monodus subterraneus	E	M	M	Huertas et al. (2002)

^a^ E: existing; M: missing; NR: not reported.

## 3 The role of microalgae in CO_2_ capture for biomass production

The increasing levels of atmospheric CO_2_, which are attributed to human activities and have caused a major shift in the global carbon cycle, have become a major global concern and a subject of research in recent years (Mondal et al., 2017). Currently, atmospheric CO_2_ constitutes approximately 77% of all greenhouse gases, making its capture crucial, even with the presence of other greenhouse gases such as hydrocarbons, sulfur dioxide, methane, and nitrogen oxides (Xu et al., 2021). The capture of atmospheric CO_2_ by microalgae during the generation of biomass along with other valuable carbon compounds, presents a promising solution to the issue of global warming (Iglina et al., 2022). Microalgae have the potential to capture and reduce atmospheric CO_2_ 10–50 times more effectively than terrestrial plants (Li et al., 2008). They also have several other advantages over terrestrial plants, such as not competing for food and feed for humans and animals, using less land and water, and possibility grow in various types of water (Osman et al., 2023b).

The cost of feedstock media used to grow microalgae is significantly impacted by the high amount of CO_2_ required, which accounts for over 50% of the cost (Doucha et al., 2005). Microalgae can efficiently absorb CO_2_ from both the atmosphere and from flue gas emissions, with capture rates up to 90% reported in open ponds (Sayre, 2010). A novel spraying absorption tower combined with an open raceway pond has demonstrated improved CO_2_ fixation efficiency of 50%, compared to 11.17% for traditional bubbling methods (Politaeva et al., 2023). As mentioned previously, microalgae have evolved unique mechanisms, such as C4, CAM, and CCM, to improve their efficiency in capturing carbon (Colman et al., 2002). However, the capture rate varies between species due to differences between the CA enzymes. A study by Bhola et al. (2014) reported that *Synechocystis aquatilis*, grown in raceways ponds with a water volume of 4,000 m^3^ and using sunlight, can absorb approximately 2,200 tons of CO_2_ y^−1^. The study by (Duarte et al., 2017) reported that the highest biomass productivity of *Chlorella fusca* LEB (25 g m^−2^ d^−1^) required 45.8 g CO_2_ m^−2^ d^−1^.

Although microalgae biomass has been commercially cultivated for more than 40 years, its entire global biomass per year was only 93,756, 87,000, and 56,465 tons in 2010, 2018, and 2019, respectively (Hamidi et al., 2023). Several reports have indicated that each 1 ton of microalgae biomass (dry weight) captured about 1.88 tons of atmospheric CO_2_ (Benemann and Oswald, 1996), while Chisti reported it as two tones (Chisti, 2007). However, it is necessary to mention that each algal strain needs to be studied independently. Based on calculations, the equivalent amounts of CO_2_ captured by cultured microalgae are around 187,500, 174,000, and 112,900 tons of CO_2_ in 2010, 2018, and 2019, respectively (Iglina et al., 2022).

There are two systems widely used in microalgae cultivation; open pond (OP) and photobioreactor (PBR). In the open systems, there are many challenges facing supplementation and capture of CO_2_, due to low fixation efficiency (usually between 10% and 40% vol.), low solubility, high cost, significant loss during culture, and poor tolerance to high CO_2_ levels (Song et al., 2019). The main advantage of using PBR for the capture of CO_2_ by microalgae is the increase in productivity according to regulated environmental factors and the optimal volume utilization. As reported in the literature, few microalgae species can tolerate CO_2_ at high levels of 70% vol. such as *Chlorella* sp. KR-1 and *Chlorella* ZY-1, 90% vol. CO_2_ such as *Chlorella vulgaris* (Li et al., 2013), while others can tolerate CO_2_ at 100% vol. CO_2_ such as *Chlorella* sp. T-1 (Zhang and Song, 2014).

The first published work on increasing microalgae biomass production by providing external CO_2_ to microalgae culture media was in the 1960s (Heubeck et al., 2007). From this date, many publications have claimed that using an external source of CO_2_ to supplement the culture medium significantly increases microalgae biomass (Judd et al., 2017). High levels (99.9%) of CO_2_ may be injected using high-purity gas cylinders. However, this is an expensive technology that can limit the use of the system, especially in open pond systems (de Assis et al., 2019), even if it is appropriate for PBR systems. To minimize costs, CO_2_ can be added to the ponds in the form of exhaust gases. To reduce the price, it would be appropriate to add CO_2_ to PBRs in the form of compressed exhaust gases to mitigate the yearly increase in CO_2_ emissions. As reported previously (Zheng et al., 2018), the possible sources of CO_2_ supplies are air, pure CO_2_ (commercial grade or purified), raw flue gas, CO_2_-containing solvents, and HCO_3_^─^. Each source has advantages and disadvantages.

Several studies (Couto et al., 2018) have claimed that adding more CO_2_ not only tends to make more carbon available for the growth of microalgae, but also enhances the assimilation of nutrients into their biomass, reducing nitrogen losses from ammonia volatilization and phosphorus precipitation, and preventing pH increases brought on by photosynthetic activity. To utilize purified CO_2_, flue gas, and solvents that contain CO_2_, it is necessary to have access to point sources of CO_2_ and terrestrial facilities appropriate for mass microalgae cultivation. Flue gas is widely available and has limited commercial value. However, it also contains impurities that may inhibit microalgae growth. While having a lower cost and less accessibility than flue gas, purified CO_2_ is the most efficient solution regarding utilization and transport. The energy required to dispense HCO_3_^─^ or CO_2_ -loaded solvents is substantially lower than the energy required for compressing and transporting the gas CO_2_. Bicarbonates are more expensive and scarcer than flue gas, even if it is purer. Furthermore, not all microalgae species can capture bicarbonates. Co-location of algae production sites with a CO_2_ collection facilities would be favorable for the use CO_2_-loaded solvents. de Assis et al. (2019) reported that since CO_2_ represents the most expensive input needed for microalgae culture in PBR systems, it is remarkable to use exhaust gases in conjunction with wastewater treatment to achieve lower input costs for microalgae. The use of CO_2_ from exhaust emissions for the growth of microalgae in PBR systems is less expensive than pure CO_2_. The cost of CO_2_ is associated with the installation, and maintenance of the PBR system, and the process of CO_2_ capture and compression, which was estimated to account for 75% of the total costs among the many expenditures associated with the procedure (Van Den Hende et al., 2012). Table 2 summarizes the advantages and disadvantages of each source of CO_2_, as reported in the literature.

**TABLE 2:** Advantages and disadvantages of several potential CO_2_ sources for microalgae production as reported in the literature (Kadam, 1997; Metz et al., 2005; Brinckerhoff 2011; Xu et al., 2014; Rubin et al., 2015; Nayak et al., 2018 **)**.

	Air	HCO_3_^─^	Commercial grade CO_2_	Purified grade of pure CO_2_	Raw flue gas	CO_2_-containing solvents
CO_2_ concentration	0.042%	0.1–5 g L^−1^ (NaHCO_3_)	> 95%	> 95%	4% – 33%	0.5 mol CO_2_/mol solvent (20%)
CO_2_ volume required	Very high	None	Low	Low	Moderate	None
Availability	Unlimited	Moderate	Low	High	High	Moderate
CO_2_ utilization	Very low	Very high	High	High	Moderate	Very high
CO_2_ cost ($ per ton)	0	380	3–55	29–111	0	10–35
Energy for compression and transportation	None	Low	Moderate	Moderate	High	Low
OP injection energy	Very high	Low	Moderate	Moderate	High	Low
PBR injection energy	None	Low	Very high	Very high	Very high	High

## 4 Factors influencing CO_2_ sequestration on an industrial scale

**FIGURE 2:**
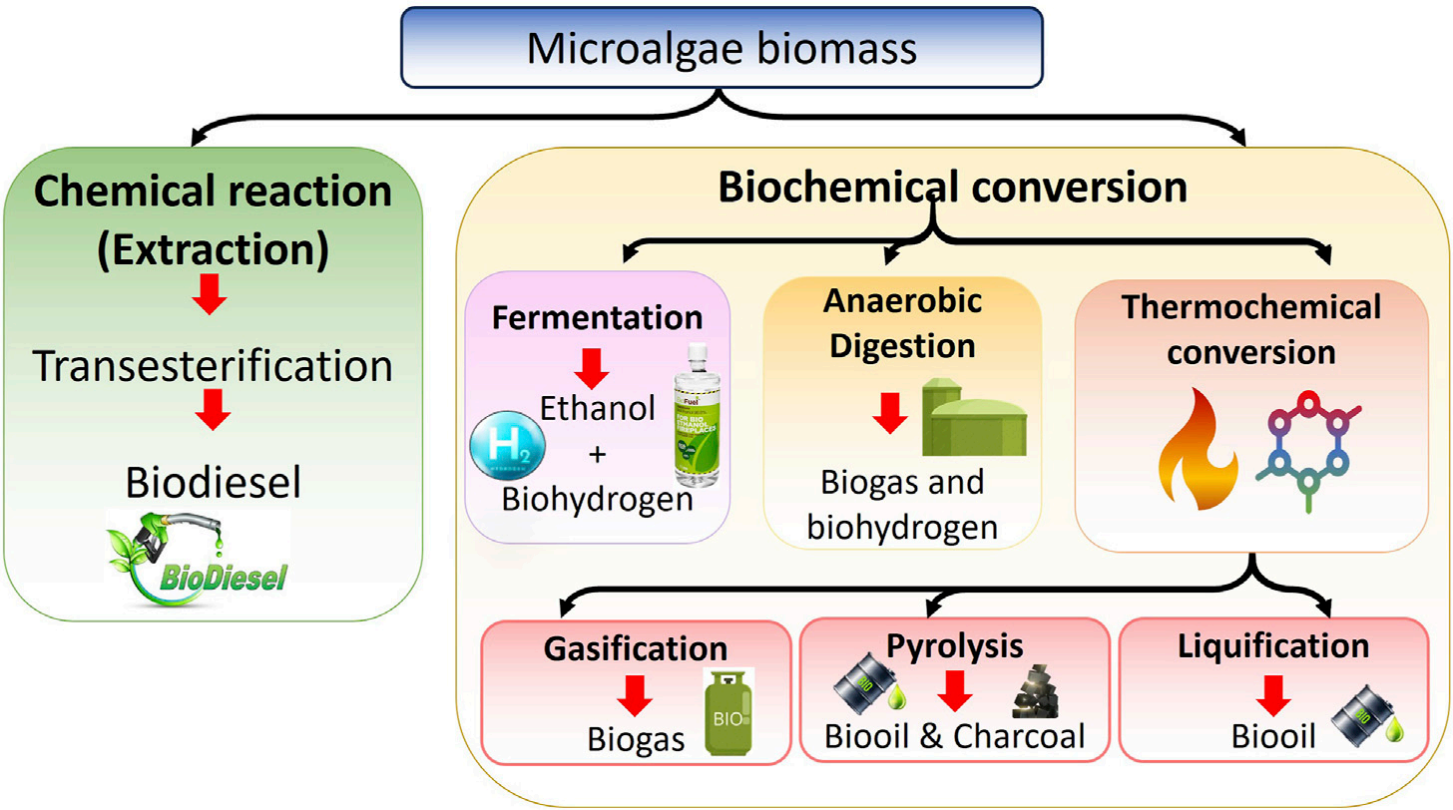
Microalgae-based biomass conversion processes for biofuel production.

The factors influencing microalgal CO_2_ sequestration are summarised in Figure 2 and will be treated in detail in the following chapters.

### 4.1 pH

Most microalgae species including *Chlorella* sp. grow optimally at a neutral to moderately alkaline pH between 7 and 9. For example, the microalgae *Chlorella stigmatophora* and *Nannochloris* sp. achieve peak growth rates at pH 8 and 7, respectively (Galès et al., 2020). During photosynthesis, microalgae take up bicarbonate ions, which in turn raises the pH of the aquatic environments (Dolui et al., 2021). This photosynthetic alkalinization hinders the dissolution of CO_2_ from the air and reduces bioavailability for further carbon fixation (Zhou et al., 2017). Low pH levels below 6–7 can also negatively impact cell metabolism and inhibit the active transport systems that allow microalgae to take up essential ions. A pH dropping too far from the ideal neutral range has been shown to reduce the growth rate of species such as *Spirulina platensis* (Zeng et al., 2011). Different cultivation strategies, such as autotrophic, mixotrophic, and photoheterotrophic, can influence the effect of pH on carbon fixation and carbohydrate accumulation in *C. vulgaris* JSC-6 (Cheng et al., 2022). Autotrophic cultivation resulted in better carbon assimilation and carbohydrate accumulation, while the assimilation of fatty acids in the mixotrophic and photoheterotrophic modes was influenced by pH. Therefore, monitoring and controlling pH is imperative when cultivating microalgae for carbon sequestration, as pH dictates the rate of photosynthesis and cellular activities that allow CO_2_ to be effectively utilized. Using species adapted to variable pH or actively managing pH through CO_2_ sparging and buffer addition enables healthy, productive algal cultures. Overall, maintaining an optimal pH range, such as around 8, can enhance carbon fixation and biomass yield in *Chlorella*. The specific pH requirements may vary depending on the species and cultivation strategy.

### 4.2 Temperature

Most microalgae species suited for CO_2_ capture are mesophilic, with an optimal growth temperature range of 25°C–45°C (Farrelly et al., 2013). Temperatures above this range reduce the solubility of CO_2_ and alter cellular enzyme functions (Prasad et al., 2021). For *Chlorella* the optimal temperature range strongly depends on the chosen species and other environmental factors. Generally, temperatures between 25°C and 35°C are considered favorable for the growth and carbon fixation of *Chlorella* (Politaeva et al., 2023)*.* Specifically, moderate temperatures cause the pivotal carbon fixation enzyme Rubisco to bind oxygen instead of carbon dioxide, resulting in photorespiration that lowers CO_2_ utilization rates by up to 30% (Zeng et al., 2011). Excessively high temperatures can damage the photosynthetic apparatus and reduce overall carbon fixation efficiency (Politaeva et al., 2023). Heat also alters the activity of other enzymes like carbonic anhydrase which interconverts CO_2_ and bicarbonate, further limiting inorganic carbon bioavailability (Ighalo et al., 2022). *Chlorella* has the ability to adapt and acclimate to different temperature conditions. Prolonged exposure to specific temperatures can trigger physiological and biochemical changes in allowing *Chlorella* to better cope with the prevailing temperature and maintain carbon fixation efficiency (Politaeva et al., 2023). Therefore, the cultivation temperature must be controlled for mesophilic species to ensure adequate CO_2_ dissolution while preventing enzyme impairment and photorespiration.

### 4.3 Irradiation

Irradiation conditions play a crucial role in regulating the photosynthetic efficiency and CO_2_ fixation capacity of microalgae. The intensity of light irradiance has a direct impact on the rate of photosynthesis and biomass productivity (Souliès et al., 2016). While higher light intensities can drive faster growth rates initially, excessive irradiance beyond the saturation point can lead to photoinhibition and decreased CO_2_ fixation. Similarly, the photoperiod, or the daily light/dark cycle duration, influences the microalgae’s ability to balance light energy absorption and dark respiration phases. Optimal photoperiods vary across species but often range from 12–16 h of light per day for efficient CO_2_ capture (Shareefdeen et al., 2023). Additionally, the spectral quality or wavelength of light can affect photosynthetic performance, with different microalgal pigments exhibiting peak absorption in specific wavelength ranges like blue, red, and green. Tailoring light sources to match the absorption spectra of the target microalgae strain has been shown to enhance biomass yields and CO_2_ fixation rates. As such, optimizing irradiation parameters like intensity, photoperiod, and spectral composition is crucial for maximizing the potential of microalgae-based CO_2_ capture systems (Sero et al., 2020).

The minimum irradiation in the range of 10–30 μmol m^-2^ s^-1^ is required for microalgae species to effectively uptake CO_2_ and convert it into biomass through photosynthesis. Light drives the first stage of carbon fixation, so sufficient photon flux density optimizes the growth rate, biomass, and CO_2_ sequestration potential of microalgae cultures. Outdoor mass cultivation systems should receive over 880 μmol photons m^-2^s^-1^ from sunlight for appreciable productivity (Hosseini et al., 2018). Additionally, the efficiency of light absorption depends on the properties of the microalgae cells themselves. Cells with a greater surface area and higher concentrations of light-harvesting pigments such as chlorophyll can absorb more useful radiation. When light becomes limited, adaptive mechanisms, like increasing pigment production become active, as demonstrated in the freshwater microalgae *Scenedesmus obliquus* (Wu et al., 2023). Another study showed a microalgal consortium of *Chlorella* sp., *Scenedesmus obliquus*, and *Ankistrodesmus* sp. could tolerate CO_2_ concentrations up to 7% in a photobioreactor system. The optimal conditions for maximizing CO_2_ removal and biomass growth were 4,000 lux (74.07 μmol m^−2^s^−1^) light intensity with a 16 h light/8 h dark cycle at 30 C. Under these conditions with a 5% CO_2_ supply, the maximum growth rate reached 0.38 per day. The synergistic action of the three species allowed efficient photosynthetic conversion of the CO_2_ into biomass. Promising results included 49.02% CO_2_ removal efficiency, 15.15% CO_2_ utilization efficiency into biomass, 101.29 gCO_2_ L^-1^ h^-1^ CO_2_ transfer rate, and 42.02 h^-1^ CO_2_ fixation rate. This demonstrates the potential of using optimized microalgal consortia in photobioreactors for effective CO_2_ mitigation by biological fixation into valuable biomass (Rinanti et al., 2014). Increasing irradiation during the culture of *Chlorella* resulted in a ∼60% increase in biomass production and a ∼7.0% increase in CO_2_ fixation ability. It was demonstrated that using bicarbonate (HCO_3_^−^) as a carbon source significantly affected cultivation, showing non-competitive inhibition under both increasing and constant photon flux density regimes. This inhibition influenced both biomass production and CO_2_ fixation rates. Another study evaluated microalgal biomass productivity and quality using different colored photobioreactors (white, blue, green) for co-cultivating *Chlorella vulgaris, Chlorella sorokiniana*, and *Scenedesmus* sp. on domestic wastewater as medium and nutrients source. The key finding was the white PBR outperformed colored PBRs, increasing microalgae productivity by factor 2.3 to 3.5. The broad spectrum transmitted by the transparent white PBR enhanced photosynthesis, growth, and accumulation of valuable metabolites compared to narrower wavelength ranges from colored reactors under sunlight exposure. Therefore, irradiation intensity, reactor transparency, and cell characteristics interact to determine how effectively microalgae can perform photosynthesis to remove CO_2_ from the environment (Khalekuzzaman et al., 2021).

### 4.4 Inorganic nutrients

To achieve optimal growth and CO_2_ fixation, microalgae require adequate amounts of macronutrients and micronutrients. Macronutrients, such as nitrogen and phosphorus, are essential for the overall growth and development of microalgae, while micronutrients, including vitamins and trace metals, are required in smaller quantities but are equally important for their optimal growth and CO_2_ fixation (Li et al., 2022). Nitrogen is an essential macronutrient for growth and metabolism. It is a key component of proteins, nucleic acids, and chlorophyll. Adequate nitrogen supply is crucial for optimal carbon fixation and biomass accumulation in microalgae. Nitrogen can be obtained by microalgae from various sources, including nitrate, ammonium, and urea. Nitrate is commonly preferred over ammonium salts for microalgae cultivation as it is more stable and less likely to cause pH shifts (Wang et al., 2024). Ammonia concentrations above 25 μM can be toxic to microalgae. However, nitrogen limitation can reduce biomass production but enhance lipid accumulation. Studies have shown that nitrogen deficiency in *Anabaena variabilis* and *Nostoc muscorum* cultures led to decreased growth rates as well as to lower levels of photosynthetic pigments which lead to reduce CO_2_ sequestration, but simultaneously increased total carbohydrate and lipid contents. Therefore, while higher nitrogen levels favor maximum biomass productivity, nitrogen depletion diverts the metabolic flux towards elevated lipid production in microalgae (Yaakob et al., 2021).

Phosphorus is another essential macronutrient for microalgae. It is a critical component of nucleic acids, ATP (adenosine triphosphate), and phospholipids, which are essential for energy transfer and membrane structure. Phosphorus can be obtained by microalgae from phosphate compounds present in the growth medium. Along with carbon, nitrogen, and phosphorus are primary nutrients needed to build microalgae biomass through photosynthesis and cellular metabolism (Cheah et al., 2015). The optimal balance depends on the species, with optimal C: N molar ratios between of 9:1 to 22:1. The molar N:P ratio varies between 1.1 and 45:1 for various microalgae (Enamala et al., 2018). Microalgae require certain vitamins for their growth and metabolism. Vitamins, such as thiamine (B1), biotin (B7), and cobalamin (B12), act as cofactors for various enzymatic reactions involved in cellular processes. Microalgae require trace elements such as manganese (Mn), zinc (Zn), copper (Cu), and molybdenum (Mo) for various metabolic processes. These trace elements act as cofactors for enzymes involved in carbon fixation and other biochemical reactions (Adamczyk et al., 2016). Iron is a cofactor for several enzymes involved in photosynthesis and respiration. It is essential for chlorophyll synthesis and electron transport. Adequate iron availability is crucial for efficient carbon fixation and chlorophyll production (Aslam et al., 2021). However, exposure to heavy metals potentially present in flue gas supplies can inhibit cultures even at concentrations as low as 1x that of emissions from coal power plants. Polyphosphate accumulation in microalgae cells can protect them from metal toxicity. Polyphosphate can bind to incoming heavy metals like copper (Cu) and cadmium (Cd), forming detoxified complexes. Studies have shown that polyphosphate-rich conditions enabled *Chlamydomonas reinhardtii* to accumulate and survive the toxic effects of Cu and Cd by sequestering these metals. Therefore, promoting polyphosphate accumulation in microalgae is a potential strategy to mitigate the inhibitory effects of heavy metal contaminants present in industrial flue gas feedstocks. Therefore, while adequate provision of both macronutrients and micronutrients is necessary to sustain healthy, productive microalgae populations for carbon capture, limiting heavy metal contamination is also crucial (Napan et al., 2015).

Microalgae species can utilize diverse waste substrates including agricultural fertilizers, livestock manure, compost extracts, food processing wastewater, anaerobic digestates, and municipal wastewater (Duan et al., 2023; El-Khodary et al., 2021). These waste streams provide nitrogen, phosphorus, and trace nutrients to sustain biomass growth. Selecting compatible cultivation substrates influences productivity given species-specific nutrient requirements, tolerance to contaminants, and optimal carbon: nitrogen ratios for balanced growth. Using agricultural runoff/wastewater streams as growth media benefits microalgae CO_2_ fixation through nutrient provision while enabling water bioremediation (Cho et al., 2020). However, high ammonia or salts from fertilizers or livestock waste can inhibit specific microalgae strains (Markou et al., 2014). Thus, the use of low-strength municipal wastewater or anaerobic digestates may improve compatibility for freshwater varieties like *Chlorella* sp. and *Scenedesmus* sp. by moderating nitrogen levels (Serejo et al., 2015). Marine and halotolerant algae conversely thrive when cultivated in high-salt substrates or with salinity adjustment using brines.

### 4.5 Dissolved oxygen

Dissolved oxygen (DO) levels play a critical role in influencing the CO_2_ fixation efficiency and overall productivity of microalgae cultivation systems. While oxygen is an essential byproduct of photosynthesis, its accumulation beyond optimal levels can have detrimental impacts on microalgal performance. High concentrations of dissolved oxygen above 25 ppm can inhibit CO_2_ fixation rates in microalgae cultures (Jiménez et al., 2003). As the accumulated DO competes with CO_2_ for the active sites of Rubisco and other enzymes involved in carbon fixation pathways (Morales et al., 2018). Additionally, DO can cause oxidative damage to cellular components through the formation of reactive oxygen species.

Studies have shown that reducing DO levels can dramatically improve carbon sequestration efficiency, with a 30-fold decrease in DO facilitating a 3-fold increase in CO_2_ fixation rate in *Chlorella* sp. (Cheng et al., 2006). These studies highlight how dissolved oxygen levels influence *Chlorella* productivity through mechanisms like photorespiration and photoinhibition, underscoring the importance of optimizing culture conditions to mitigate such detrimental effects. The mechanisms underlying the inhibitory effects of high DO on CO_2_ fixation are not fully understood but may involve damage to photosynthetic machinery, competition for enzyme active sites, and altered carbon partitioning pathways under oxidative stress conditions.

Therefore, regulating and maintaining optimal dissolved oxygen levels is critically important not only for ensuring the overall health and growth of microalgae cultures but also for maximizing their efficiency of CO_2_ biofixation productivity and efficiency. Strategies such as controlled aeration, gas sparging, and the selection of microalgae species adapted to high oxygen tolerance can help mitigate the inhibitory effects of elevated DO. However, understanding and managing both DO and CO_2_ levels is crucial for optimizing microalgal CO_2_ biofixation rates in carbon capture and utilization (CCU) approaches (Gao et al., 2022).

### 4.6 CO_2_ sources and - concentration

The efficacy of CO_2_ biofixation by microalgae depends substantially on the growth substrates and carbon sources employed in the cultivation system. In addition to inorganic carbon sources such as bicarbonate (HCO_3_^−^) and CO_2_ (Politaeva et al., 2023) also organic carbon sources, such as glucose and acetate, can be used by microalgae including *Chlorella* (Politaeva et al., 2023).

CO_2_ concentrations in the aeration gas below 0.5% vol. limit microalgal growth and CO_2_ utilization due to poor solubility in water and low substrate affinity of carbon fixation enzymes such as Rubisco (Mustafa et al., 2020). However, high levels above 6%–12% vol. can also reduce growth by altering the CCM, which converts dissolved CO_2_ into more bioavailable bicarbonate (HCO_3_^−^) (Zeng et al., 2021). It has been reported that, elevated CO_2_ concentrations above 30 g m^-3^ cause a 30% loss in biomass productivity in *Chlorella vulgaris*, indicating a negative effect of high dissolved CO_2_ levels (Kazbar et al., 2019). Specifically, excessive CO_2_ inhibits the enzyme carbonic anhydrase, which catalyzes the interconversion between CO_2_ and HCO_3_^−^, resulting in limited inorganic carbon for fixation (Abomohra et al., 2023). A comparative study (Koh et al., 2023) analyzed the transcriptomic changes in *Chlorella* sp. ABC-001 under ambient air and high CO_2_ conditions. The study aimed to understand the molecular mechanisms driving carbon fixation and lipid accumulation in microalgae. The results revealed significant transcriptional changes in response to different CO_2_ concentrations, indicating the importance of CO_2_ availability in regulating pathways such as carbon fixation, photosynthesis, and possibly stress responses. Recent work has also demonstrated that CO_2_ levels around 15% paired with adequate gas transfer rates maximize carbon fixation in mixotrophic microalgae cultivation (Ahn et al., 2022). Another study found carbonic anhydrase enzyme levels nearly diminished in *Chlorella* cells grown under 15% vol. CO_2_, indicating direct CO_2_ permeation into cells without needing the CO_2_-concentrating mechanism (CCM). The estimated minimum intracellular CO_2_ concentration required by Rubisco in this strain ranged from 80–192 μM. Bypassing the energy-intensive CCM under high CO_2_ saved ATP for carbon fixation pathways. Notably, Rubisco gene expression was 16.3 times higher at 15% vol. CO_2_ *versus* air, while transcript levels of other key carbon fixation genes were also upregulated under elevated CO_2_, while high CO_2_ levels over 15% diminished the algal growth at all. Therefore, there is an ideal CO_2_ dosage range for microalgae lying between substrate limitation and toxicity thresholds where growth and assimilation efficiencies peak (Park et al., 2021).

Flue gases are an important source of CO2. However, their pretreatment is crucial as microalgae are susceptible to damage from contaminants like sulfur oxides (SO_x_), nitrogen oxides (NO_x_), and particulates, despite having protective cell walls (Khoo et al., 2020). Desulfurization reduces SO_x_ levels below 60 ppm, which is essential for microalgae growth, while denitrification lowers NO_x_. De-dusting flue gas by scrubbing particulates is another beneficial pretreatment, especially when using emissions directly from combustion sources like power plants (Bhola et al., 2014). Flue gas pretreatment can be achieved through various methods, including wet scrubbing, dry sorbent injection, and catalytic converters. Advanced pretreatment technologies, such as membrane separation and ionic liquids, are also being explored for efficient contaminant removal (Isosaari et al., 2019). Optimal preconditioning methods can make microalgae cultivation systems more productive and cost-effective for biological carbon capture (Thomas et al., 2016).

## 5 *Chlorella* as microalga model for biomass production

The green microalga *Chlorella* sp. is renowned for its remarkably rapid growth rate, making it one of the fastest-growing algae species (Bouyam et al., 2017). This versatile alga is extensively cultivated and utilized in a wide range of applications, including food and feed production, wastewater treatment, and flue gas remediation (Najm et al., 2017) *Chlorella sp*. possesses an exceptional ability to thrive under diverse conditions, favoring either the autotrophic, heterotrophic, or mixotrophic growth mode. This adaptability contributes to its widespread utilization across various industries. Several *Chlorella* species, including *C. pyrenoidosa, C. vulgaris, C. lewinii,* and *C. sorokiniana*, exhibit the capacity to accumulate carbohydrates. In addition to biomass production *Chlorella* can produce high-value by-products such as carotenoids, vitamins, and fatty acids, making them attractive candidates for various industrial applications (Ngangkham et al., 2012). The robustness, high growth rate, and high content of neutral lipids in *Chlorella* sp. make it a promising candidate for bioenergy production. To reduce costs and enhance the economic viability of algal biomass production, indoor photobioreactor (PBR) systems have been developed for high-density cultivation. These systems offer controlled environmental conditions, optimizing growth and productivity (Bhushan et al., 2023). The integration of renewable energy sources and carbon capture technologies further contributes to the sustainability and environmental benefits of *Chlorella* sp. cultivation (Amaral et al., 2020).

*Chlorella* sp. was selected as a model for CO_2_ capture due to its high biomass productivity and large biochemical profile, which includes various carbon pathways, such as CMM, C4, and CAM. It can tolerate high levels of CO_2_, up to 70%, 95% (Li et al., 2013), and 100% vol. (Zhang and Song, 2014), and does not require high pH values, making it a suitable option for CO_2_ capture compared to other species like as Arthrospira platensis (Cai et al., 2021) Notable species of *Chlorella* include *Chlorella vulgaris*, *Chlorella sorokiniana*, and *Chlorella protothecoides*, all of which are recognized for their efficiency in CO_2_ capture and biomass production. According to the FAO statistics report (FAO, 2020), in 2019, *Chlorella vulgaris* accounted for 0.008% of the world’s total microalgae production, with a production volume of 4.77 tons. This made it the third most produced microalgae species globally. However, it is important to note that this report did not include other species within the *Chlorella* genus. The *Chlorella* genus has a multitude of commercial applications, including production of food supplements (Couto et al., 2022), pharmaceuticals (Lamare and Chaurasia, 2022) glycolipids (Yamashita et al., 2022), PUFA (Toumi et al., 2022), biodiesel (Moradi and Saidi, 2022), biohydrogen (Jimenez-Llanos et al., 2020a), and bioplastic (Nanda, 2022). Additionally, *Chlorella* is used in aquaculture (Ranglová et al., 2022) and, wastewater purification (Kumari et al., 2022). The CO_2_ capture rate (g L^−1^ d^−1^) and removal efficiency (%) of *Chlorella* species are influenced by various cultivation conditions, such as CO_2_ volume, temperature, pH, and light intensity, as indicated in Table 3.

**TABLE 3:** Reported CO_2_ removal capacity and capture rate of selected *Chlorella* species in PPR under different growth conditions.

Chlorella species	Biomass	CO_2_ removal efficiency (%)	CO_2_ capture rate (g L^−1^ d^−1^)	Cultivation Conditions				
				CO_2_ (vol%)	Temp (°C)	Light intensity (Lux)	pH	References
C. vulgaris	23.5 (10^6^ cells m L^−1^)	18	–	30	30	1800	7.2	Sadeghizadeh et al. (2017)
C. vulgaris	18.3 (10^6^ cells m L^−1^)	18	2.22	–	30	3,783	–	Sadeghizadeh et al. (2017)
C. vulgaris	-	40	0.51	10–20	8	4,540	8.2	Sadeghizadeh et al. (2017)
C. vulgaris	-	–	0.25–1.7	0.03	18	6,000	8	Sadeghizadeh et al. (2017)
Chlorella sp.	3.461 (g L^−1^)	10	–	10	26	16,000	–	Chiu et al. (2009)
Chlorella sp.	2.369 (g L^−1^)	5	0.35	5	–	5,400	7.1	Chiu et al. (2009)
C. vulgaris	1.1–1.9 (g L^−1^)	18	2.66	25	25	3,600	7–8.2	Fan et al. (2008)
Chlorella sp.	5.77 (g L^−1^)	10	–	10	25	4,050	6	Yue and Chen (2005)

As with any microalgae species, the methods and conditions for cultivating *Chlorella* vary based on the intended use of its biomass. Valdovinos-García et al. (2021) conducted a techno-economic study of harvesting and drying *Chlorella* cultivated in tubular photobioreactors and found a biomass production of 82.45 tons ha^−1^ y^−1^ (22.66 g m^2^ d^−1^) with estimated CO_2_ capture of 148.4 148.4 tons ha^−1^ y^−1^. In another study (James and Al-Khars, 1990), *Chlorella* sp. MFD-1 cultured in airlift PBR produced a biomass of 109–264 g m^−3^ d^−1^. Another study by Hossain et al. (2019) reported that the biomass productivity of *Chlorella* was around 56 tones ha^−1^ y^−1^, with a capture of 36.3 tons CO_2_ ha^−1^ y^−1^. On the other hand, the study by Bhola et al. (2014), and Bai et al. (2017) showed that the average biomass production of microalgae cultivated in open ponds was approximately 175, 280, and 300 tons ha^−1^ y^−1^, respectively, which could potentially lead to the capture of 329, 525, and 564 tones CO_2_ ha^−1^ y^−1^, respectively. These data suggest that the production of biomass is largely influenced by the cultural technology used. Improved and advanced technologies result in increased biomass. Min et al. (2011) conducted a pilot-scale study on the bioremediation efficiency of *Chlorella* sp. grown in a 1200 L tubular photobioreactor using wastewater, resulting in a biomass production range of 17.7–34.6 g m^−2^ d^−1^. This shows that *Chlorella* sp. is a promising option for CO_2_ capture compared to terrestrial plants, taking into account the technologies used for cultivation, harvesting, and drying.

Chiu et al. (2009) conducted a study on the culture of marine *Chlorella* sp. in a 750 mL photobioreactor under controlled conditions and a temperature of 26°C, continuous cool-white, fluorescent light (intensity of 300 μmol m^−2^ s^−1^), using F/2 medium. The study examined the effect of different CO_2_ levels (2, 5, 10, and 15% vol.) on average biomass productivity, CO_2_ capture, and efficiency. The results showed that the average biomass productivity ranged from 0.76 to 0.87 g L^−1^ after 8 days of cultivation, while the CO_2_ capture and efficiency ranged from 0.261 g h^−1^ and 58% to 0.573 g h^−1^ and 16%, respectively. The study concluded that using an advanced multiple photobioreactor system can increase the efficiency of *Chlorella* sp. in CO_2_ capture. Vo et al. (2018) conducted a study on the effect of different N/P ratios (10/1, 15/1, 20/1, and 25/1) on the biomass productivity of *Chlorella* sp. in a 2-working bubble column photobioreactor with a total volume of 8 L. The study was conducted under controlled conditions of temperature (29°C ± 2°C), continuous irradiation (3,000 Lux), air and air mixture (2 and 4 L min^−1^), and injected CO_2_ at a level of 0.2 L min^−1^. The optimum N/P ratio (15/1) was found to result in biomass productivity of 3.568 g L^−1^, a CO_2_ capture efficiency of 28%, and a CO_2_ removal rate of 68.9 mg L^−1^ h^−1^. Table 4 summarizes some published studies on microalgae biomass cultivated under different types of photobioreactors.

**TABLE 4:** Biomass production of some *Chlorella* species cultured in different PBR systems.

*Chlorella species*	*PBR Types*	Biomass production	References
*Chlorella* sp	Airlift	109–264 g m^−3^ d^−1^	James and Al-Khars (1990)
*C. vulgaris*	Airlift	0.28–0.89 g L^−1^ d^−1^	Hanagata et al. (1992)
*C. vulgaris*	Airlift	0.124 g L^−1^ d^−1^	Ammar (2016)
*C. vulgaris*	Airlift	460 mg L^−1^ d^−1^	Ren et al. (2017)
*C. pyrenoidosa*	Airlift	0.37 g L^−1^ d^−1^	Tan et al. (2014)
*C. vulgaris*	LED-based PBR	2.11 g L^−1^ d^−1^	Fu et al. (2012)
*C. sorokiniana*	Light-path-panel PBR	2.1 g L^−1^	Cuaresma Franco et al., 2024
*C. vulgaris*	Rotating float-plate PBR	3.35 g m^−2^	Melo et al. (2018)
*C. sorokiniana*	Flat plate	469 mg L^−1^ d^−1^	Do et al. (2022)
*C. zofingiensis*	Flat plate	58.4 mg L^−1^ day^−1^	Feng et al. (2011)
*C. vulgaris*	Flat plate	0.045 g L^−1^ h^−1^	Satoh et al. (2001)
*Chlorella* sp	Bubble column	3.5 g L^−1^	Bui et al. (2018)
*C. vulgaris*	Bubble column	1.41 g L^−1^	De Morais and Costa (2007)
*C. minutissima*	Bubble column	1.65 g L^−1^	Sharma et al. (2016)
*C. vulgaris*	Column	81.67 mg L^−1^ d^−1^	Bamba et al. (2015)
*C. vulgaris*	Column	0.28–0.52 g L^−1^	Lam and Lee (2014)
*C. sorokiniana*	Column	10.22 g m^−2^ d^−1^	Béchet et al. (2013)
*Chlorella* sp	Tubular	21.5 g m^−2^ d^−1^	Watanabe and Saiki (1997)
*C. pyrenoidosa*	Tubular	1.83–2.10 g L^−1^	Tan et al. (2021)
*Chlorella* sp	Tubular	17.7–34.6 g m^−2^ d^−1^	Min et al. (2011)
*Chlorella* sp	Tubular	22.66 g m^−2^ d^−1^	Valdovinos-García et al. (2021)

The production cost of microalgae biomass has significantly decreased due to the utilization of knowledge from granted patents (Mohamed, 2018), along with practical expertise, know-how, and field experiences. These tools simplify the processes of culture, harvesting, drying, and extraction. Our objectives align with the findings of Acién et al. (2012) who performed a cost analysis of producing high-value products from *S. almeriensis*. Over 2 years at a small scale (0.04 ha), *S. almeriensis* was grown in 10 m^3^ tubular PBRs. The resulting annual production capacity, photosynthetic efficiency, and production cost were 3.8 tons per year (90 tons ha^−1^ y^−1^), 3.6%, and 69 € kg^−1^, respectively. They concluded that increasing production capacity leads to a decrease in production cost. Furthermore, large projects and facilities have the potential to produce more than 200 tons ha^−1^ y^−1^ and they often have lower labor and consumption costs. Acién et al. (2012) also suggested using flue gases and wastewater as sources of external CO_2_ or carbon. In conclusion, they found PBRs to be more attractive and productive than OP systems but recommended reducing the fixed and operational costs to make them more comparable to those of OP systems. They also advised implementing new PBR technologies. However, this study was conducted over a decade ago, and PBR technologies have since advanced, which may have changed the production costs.

## 6 Microalgae as a feedstock for bioenergy

Through biofixation, being the resulting biomass rich in carbohydrates, lipids, and other compounds can then be converted into sustainable biofuels via various thermo-chemical and biological routes. Biofuels offer several advantages as they are environmentally friendly, non-toxic, and can serve as an alternative to fossil fuels. Current efforts are focused on effectively utilizing various waste streams as feedstocks for commercial biofuel production. With the limitations associated with first and second-generation biofuels from food crops and lignocellulosic biomass, microalgae have emerged as a promising third-generation biofuel source to replace fossil fuels (Abomohra and Elshobary, 2019). Species like *Chlorella* sp., *Botryococcus braunii, Dunaliella primolecta*, and *Nannochloropsis* sp. can produce substantial amounts of hydrocarbons and lipids that can be converted to biofuels, in addition to their biomass. These microalgae also synthesize other commercially valuable compounds such as polysaccharides and carotenoids. Furthermore, microalgae have the ability to grow on diverse media, and their biomass is abundantly available. However, a major challenge in microalgal biofuel production is the inherently low lipid content of the cells and their small size, which makes the harvesting process extremely costly and difficult to implement at a commercial scale. Consequently, developing cost-effective harvesting strategies is one of the critical barriers hindering the widespread marketability and economic viability of microalgae-based biofuel production systems. Furthermore, the combination of highly scalable microalgae productivity coupled with carbon capture, nutrient recycling, and value-added biofuel coproduction makes microalgal biorefinery systems uniquely promising and drives further process advancement (Goh et al., 2019). Figure 2 schematically illustrates overall biofuel production processes.

### 6.1 Biodiesel production

Microalgae can accumulate significant amounts of lipids, making them a promising feedstock for biodiesel production (Chisti, 2007). They can synthesize and store lipids, primarily in the form of triacylglycerols (TAGs), which can account for up to 60% of their dry cell weight under certain cultivation conditions. The ability of microalgae to accumulate high levels of lipids is attributed to several factors, including their simple cellular structure, rapid growth rate, and the ability to modulate their metabolism in response to environmental conditions (Elshobary et al., 2022; Osman et al., 2023b). For example, when subjected to stress conditions such as nutrient deprivation, high light intensity, or temperature changes, some microalgal species can divert their metabolic pathways towards increased lipid biosynthesis and accumulation as a survival mechanism. This lipid-rich biomass can be used as a feedstock for the production of biodiesel through transesterification, a process that converts the TAGs into fatty acid methyl esters (FAMEs), which are the main components of biodiesel. Compared to traditional feedstocks like vegetable oils or animal fats, microalgal lipids offer several advantages, such as higher productivity per unit area, the ability to grow on non-arable land, and the potential to utilize waste streams (e.g., CO_2_ and wastewater) as nutrient sources (Abomohra and Elshobary, 2019).

Microalgae oil, rich in fatty acids like linoleic (C18:2), linolenic (C18:3), and oleic acid (C18:1) can be extracted and converted to biodiesel through transesterification reactions with alcohols like methanol. Microalgae species, including *Nannochloropsis*, *Chlorella,* and *Schizochytrium* contain 20%–77% lipid that transesterified into monoalkyl esters comparable to conventional petroleum-derived diesel (Okeke et al., 2022). However, the rigid cellulose-containing walls of microalgae resist solvent penetration during extraction. Various pretreatment methods like microwave irradiation, ultrasonication, or chemical disruption using acids/bases permeabilize the cells to improve oil recovery (Malekghasemi et al., 2021). *In situ* approaches also directly transesterify wet microalgae biomass containing up to 40% moisture into fatty acid methyl esters (FAMEs), overcoming the barrier of high water content that is typically inhibitory to biodiesel synthesis (Nguyen et al., 2020). Integrating biodiesel generation with microalgae cultivation can provide sustainable transportation fuels while recycling carbon emissions into growth substrate.

### 6.2 Biooil production

Biooil production from algal biomass is an alternative process for converting microalgal biomass into liquid fuel through thermochemical conversion techniques, such as pyrolysis or liquefaction. Unlike biodiesel production, which focuses on extracting and transesterifying the lipids present in microalgae, biooil production aims to convert the entire algal biomass into a complex liquid mixture of oxygenated hydrocarbons, known as biooil or biocrude (Mathimani et al., 2019).

The first method is pyrolysis. Pyrolysis is the thermal decomposition of algal biomass in the absence of oxygen or any other gaseous oxidizing agent. The process involves heating the dried algal biomass to temperatures ranging from 400°C to 600°C, resulting in the formation of biooil, biochar (solid residue), and non-condensable gases. The biooil obtained is a complex mixture of oxygenated hydrocarbons, including phenolic compounds, acids, alcohols, and other organic compounds (Vuppaladadiyam et al., 2023).

The second method is hydrothermal liquefaction (HTL). HTL involves the conversion of algal biomass into biooil through thermochemical reactions in an aqueous environment at elevated temperatures (300°C–350 C) and pressures (5–25 MPa). The high pressure and temperature conditions facilitate the depolymerization and decomposition of the algal biomass, resulting in the formation of biooil, solid residue, and an aqueous phase containing dissolved products. The biooil produced through HTL typically has a higher energy density and lower oxygen content compared to pyrolysis biooil. The biooil obtained from these processes can be upgraded through various techniques, such as catalytic hydrotreating, hydrocracking, or esterification, to improve its quality and stability for use as a transportation fuel or as a feedstock for the production of chemicals and materials (Gollakota et al., 2018).

Bio-oil yields around 75% on a weight basis and contains a complex mixture of oxygenated hydrocarbons like organic acids, aldehydes, ketones, and phenols with promising applications for heat, power, and transportation (Chen et al., 2015). Catalytic pyrolysis augments bio-oil quality through deoxygenation and secondary hydrocarbon reformation mediated by catalysts including zeolites and supported metal catalysts (Li et al., 2021). To potentially improve the viability of the petrochemical sector, hydroprocessing using sulfided Ni- and Co-based catalysts also reduces nitrogen while increasing carbon chains within the algae bio-oil (Babatabar et al., 2022). Microalgae biooil could ultimately provide a sustainable replacement for fossil oil-derived petrochemicals.

### 6.3 Bioethanol

Bioethanol production from algal biomass is an alternative approach to utilizing microalgae as a feedstock for biofuel production. Bioethanol is a renewable fuel that can be produced through the fermentation of carbohydrates present in biomass. Microalgae can accumulate significant amounts of carbohydrates, primarily in the form of starch or glycogen, making them a potential source for bioethanol production (Lakatos et al., 2019). Species such as *Chlamydomonas reinhardtii* and *Chlorella vulgaris* accumulate high levels of glycogen and starch, while cellulose is abundant in cell walls (Fivga et al., 2019). *Chlorella* accumulates high levels of starch and other glycans like glucans and mannans in addition to cellulosic cell walls. However, these complex polysaccharides cannot be directly fermented and instead undergo hydrolysis to convert the carbohydrates (e.g., starch, glycogen) into fermentable sugars (e.g., glucose, maltose). This can be achieved through enzymatic hydrolysis using amylases or acid hydrolysis using dilute or concentrated acids. Methods such as microwave or ultrasound pretreatments help break down the *Chlorella* cell walls, improving accessibility (Chen and Yang, 2021). Acid or alkaline pretreatments help break down the cell walls, while commercial enzyme cocktails containing amylases, cellulases, and pectinases depolymerize glycans into hexose/pentose sugars like glucose and xylose (Phwan et al., 2018). The released sugars then undergo fermentation by organisms like *Saccharomyces cerevisiae* or *Zymomonas mobilis* to produce ethanol (5%–15% v/v). The fermented broth is distilled to separate the bioethanol from the residual biomass and other components. Further purification steps, such as dehydration or molecular sieve adsorption, may be employed to obtain anhydrous bioethanol. Optimizing the processing pathways and genetics of microalgae strains could continue to improve the economic viability of microalgal ethanol production. *Chlorella vulgaris* can accumulate up to 37% of its dry weight as starch, making it a promising feedstock for bioethanol production after hydrolysis using fungal hydrolysis enzymes (Monjed et al., 2021). *Chlamydomonas reinhardtii* has been studied extensively for its ability to accumulate starch and produce bioethanol through fermentation (Choi et al., 2010). *Arthrospira platensis* can accumulate glycogen up to 65% of its dry weight, which can be hydrolyzed and fermented for bioethanol production (Kusmiyati et al., 2020). *Scenedesmus obliquus* can accumulate significant amounts of carbohydrates (up to 50% of its dry weight) and has been investigated for bioethanol production (Yirgu et al., 2023). It is important to note that the production of bioethanol from microalgae is still in the research and development phase, and various challenges, such as improving the carbohydrate content, optimizing the pretreatment and hydrolysis processes, and reducing production costs, need to be addressed for large-scale commercial viability.

### 6.4 Biogas

Biogas can be produced from algal biomass through anaerobic digestion, which is a biological process that breaks down organic matter in the absence of oxygen. Algal biomass, particularly after being used for other applications like extracting lipids or carbohydrates, contains a significant amount of residual organic matter that can be utilized for biogas production (Alzate et al., 2014). The algal biomass may need to undergo pretreatment processes to improve its biodegradability and accessibility for the anaerobic digestion process. Pretreatment methods can include mechanical (e.g., milling, ultrasound), chemical (e.g., acid, alkali), or biological (e.g., enzymatic) techniques. The pretreated algal biomass is fed into an anaerobic digester, where it is broken down by a consortium of microorganisms in the absence of oxygen. The anaerobic digestion process typically occurs in four stages: hydrolysis, acidogenesis, acetogenesis, and methanogenesis. The end product of anaerobic digestion of microalgae by methanogenic archaea and bacteria produces biogas, which contains 50%–70% methane plus 30%–50% carbon dioxide and trace gases like hydrogen sulfide and ammonia (Pavithra et al., 2020). Hydrolysis first breaks down lipids, proteins, and carbohydrates into simple monomers. Acidogenic bacteria then produce volatile fatty acids that are converted to acetic acid, hydrogen, and CO_2_. Finally, methanogens like *the* Archaea bacterium *Methanothrix* produce methane using the acetyl-CoA pathway coupled with hydrogen oxidation. However, multiple factors influence the methane productivity from microalgae including the distribution of macromolecules for digestion, the molar carbon: nitrogen (C: N) ratio optimally around 20–25:1, as well as rigid cell wall lysis. Applying preprocessing like ultrasonication to fragment cell walls can enhance biogas yields from *Chlorella vulgaris* and other species by up to 40% (Park et al., 2021).

Biogas production from algal biomass offers several advantages, including, algal biomass residues from other processes can be valorized for biogas production, contributing to a more sustainable and circular biorefinery approach. Furthermore, digested residue (digestate) from the anaerobic digestion process can be used as a nutrient-rich fertilizer or soil amendment, promoting nutrient recycling. However, there are also challenges associated with biogas production from algal biomass, such as the need for efficient pretreatment methods, optimization of the anaerobic digestion process for algal feedstocks, and the potential presence of inhibitory compounds or contaminants that can affect the microbial communities involved in the process. Several studies have investigated the potential of different microalgal species, such as *Chlorella, Scenedesmus*, and *Spirulina*, for biogas production through anaerobic digestion (Zabed et al., 2020). Ongoing research aims to improve the efficiency and economics of this process, making it a viable option for valorizing algal biomass and producing renewable energy.

### 6.5 Biohydrogen

*Chlorella* demonstrates high potential for biohydrogen production, leveraging existing biomass facilities in countries like the United States, Germany, and Japan. Through co-production of valuable by-products alongside biohydrogen, a flexible biorefinery approach could foster a sustainable bio-economy (Jimenez-Llanos et al., 2020a). Various *Chlorella* species, including *C. fusca, C. homosphaera, C. pyrenoidosa, C. vacuolata, C. vulgaris var. vulgaris, C. lewinii, C. salina, C. sorokiniana, C. protothecoides*, and *Parachlorella kessleri* (formerly *C. kessleri*), accumulate significant endogenous carbohydrates under nutrient limitation, leading to impressive hydrogen production rates. These species also produce high-value commercial by-products such as vitamins, carotenoids, glycerol, mycosporine-like amino acids, unsaturated fatty acids, lectins, anti-freeze proteins, glycoproteins, butylated hydroxytoluene, specific polysaccharides, and glutathiones (Jimenez-Llanos et al., 2020a).

*Chlorella* biomass possesses unique characteristics that qualify it for biohydrogen production: a. High biohydrogen production potential: *Chlorella* biomass, particularly species like *Chlorella vulgaris* and *Chlorella sorokiniana*, show significant biohydrogen production potential due to the presence of specific enzymes such as [FeFe]-hydrogenase (Wang et al., 2021). b. Unique H-cluster structure: *Chlorella* biomass features a distinctive H-cluster structure in its [FeFe]-hydrogenase, enhancing catalytic activity for biohydrogen production compared to other hydrogenases (Wang et al., 2021). c. Efficient photosynthesis: Known for high photosynthetic efficiency, *Chlorella* efficiently converts solar energy into biomass, crucial for biohydrogen production (Velmozhina et al., 2023). d. Tolerance to adverse conditions: *Chlorella* cells exhibit resilience to adverse conditions like high light intensity, temperature fluctuations, and nutrient limitations, ensuring consistent growth and biohydrogen production (Velmozhina et al., 2023).

Biohydrogen production from *Chlorella* spp. can be achieved through various methods including direct biophotolysis, indirect biophotolysis, and dark fermentation. Direct biophotolysis utilizes photosynthesis to split water molecules and generate hydrogen gas. Some *Chlorella* species split water in photosystem II, transferring excited electrons to Photosystem I where hydrogenase enzymes produce H_2_, though challenges with oxygen sensitivity limit efficiency (Lam et al., 2019). Indirect biophotolysis: Indirect biophotolysis involves usage of stored energy molecules like starch for hydrogen production, separating oxygenic photosynthesis from anaerobic hydrogen generation to overcome oxygen sensitivity. Dark Fermentation: In dark fermentation, microorganisms convert organic substrates into hydrogen and other by-products in the absence of light. *Chlorella* spp. can undergo dark fermentation with nitrogen-fixing bacteria like *Klebsiella* sp. and *Clostridium* sp., utilizing glycolysis and citric acid pathways to produce hydrogen (El-Sheekh et al., 2023; Elshobary et al., 2024). Dark fermentation offers advantages including high H_2_ production rates, utilization of organic substrates, and potential for valuable by-products, making it suitable for wastewater treatment and biofuel production (El-Sheekh et al., 2023; Elshobary et al., 2024).

## 7 Conclusion and future perspectives

Mitigating climate change by reducing atmospheric CO_2_ levels is a critical global challenge. While CO_2_ capture projects are environmentally beneficial, they are often viewed as low-profit and high-risk ventures requiring substantial investment. To meet 2050 climate goals, the Global CCS Institute estimates around 2,000 commercial CO_2_ capture projects must be deployed annually at $655–1,280 billion (Rassool, 2021). Encouraging private sector participation through attractive carbon pricing mechanisms like taxes or emissions trading can incentivize investment in CO_2_ capture technologies. Tax exemptions and low facility costs may also help facilitate commercial viability. Industries with high CO_2_ emissions could potentially be required to finance and develop capture projects. Microalgae, especially the *Chlorella* genus reviewed here, are emerging as a crucial tool for CO_2_ capture and conversion into valuable products like biohydrogen. Their carbon concentrating mechanisms allow 10–50x higher CO_2_ fixation than terrestrial plants. However, fully realizing microalgae’s potential requires continued scientific, commercial, and technical innovation (Iglina et al., 2022). The capacity of biohydrogen production emphasizes *Chlorella’s* versatility as an integrated biorefinery feedstock. Combining microalgae’s exceptional growth with waste resource recycling enables sustainable, circular bioeconomy. Overall, this review underscores microalgae-based CO_2_ capture as a promising solution, but large-scale implementation will require multidisciplinary advances in biological understanding, bioprocess engineering, and supportive policy mechanisms to make these technologies economically viable for achieving climate targets.
